# Chemokine Receptor-Targeted Therapies: Special Case for CCR8

**DOI:** 10.3390/cancers14030511

**Published:** 2022-01-20

**Authors:** Bernhard Moser

**Affiliations:** Division of Infection & Immunity, Henry Wellcome Building, School of Medicine, Cardiff University, Cardiff CF14 4XN, UK; moserb@cardiff.ac.uk

**Keywords:** immunotherapy, cancer, chemokine, CCR8, regulatory T cells, Tregs

## Abstract

**Simple Summary:**

Antibodies directed at so-called immune checkpoint molecules represent a substantial improvement in cancer therapy. These biological reagents highlight the exquisite interplay between cancer and our own immune system. Cancer progression is enabled by establishing a compartment of suppressive immune cells within the tumor. Therefore, elimination of these suppressor cells is an attractive strategy to augment beneficial anti-tumor immunity and to further improve the newly established immune checkpoint therapy. This review focuses on CCR8, a chemokine receptor highly expressed on suppressive immune cells, and its potential value as a novel target in cancer therapy.

**Abstract:**

Immune checkpoint blockade inhibitors (CBIs) targeting cytotoxic T lymphocyte associated protein-4 (CTLA-4) and program death receptor-1 (PD-1) or its ligand-1 (PD-L1) have transformed the outlook of many patients with cancer. This remarkable progress has highlighted, from the translational point of view, the importance of immune cells in the control of tumor progression. There is still room for improvement, since current CBI therapies benefit a minority of patients. Moreover, interference with immune checkpoint receptors frequently causes immune related adverse events (irAEs) with life-threatening consequences in some of the patients. Immunosuppressive cells in the tumor microenvironment (TME), including intratumoral regulatory T (Treg) cells, tumor-associated macrophages (TAMs) and myeloid-derived suppressor cells (MDSCs), contribute to tumor progression and correlate with a negative disease outlook. Recent reports revealed the selective expression of the chemokine receptor CCR8 on tumor Treg cells, making CCR8 a promising target in translational research. In this review, I summarize our current knowledge about the cellular distribution and function of CCR8 in physiological and pathophysiological processes. The discussion includes an assessment of how the removal of CCR8-expressing cells might affect both anti-tumor immunity as well as immune homeostasis at remote sites. Based on these considerations, CCR8 appears to be a promising novel target to be considered in future translational research.

## 1. Introduction

Numerous chemokines are involved in the development of cancer, reflecting the microcosmos of immune cells and tumor-associated tissue cells required for the establishment of tumors, their growth and, eventually, their dissemination to distant organs. The principal function of chemokines is the induction of cell migration via binding to chemokine receptors expressed on target cells [1,2,3]. Eighteen human chemokine receptors, each specific for one or several of a total of 40 human chemokines, transduce signals via the heterotrimeric G-protein pathways involving phospholipase C-mediated second messengers and subsequent activation of protein kinases and small GTPases [4,5]. Cell migration is an ongoing process of sensing extracellular chemokine gradients and, therefore, chemokine receptor signaling is quickly terminated. Following internalization, free chemokine receptors are then re-expressed on the leading edge of moving cells. In addition to the prototype chemokine response, some chemokines also contribute to cell survival, differentiation and proliferation via the NFkB and/or ERK/MEK pathways. This second category of cell responses is most notable in the combination with synergistic, chemokine-unrelated stimuli. Selected chemokine receptors present on individual immune cells work, in combination with adhesion molecules, as address codes that define their target tissue [1,2,3]. Broadly speaking, the superfamily of chemokines can be broken down into two categories: (1) homeostatic chemokines that are produced under steady-state (non-inflamed) conditions and control everyday immune cell traffic at distinct locations, including primary and secondary lymphoid tissues as well as healthy organs, and (2) inflammatory chemokines, produced locally in response to inflammatory stimuli, that mediate the recruitment of effector immune cells to sites of infection and chronic inflammation [6,7].

## 2. Complex Interactions of Chemokines with Tumor-Associated Immune and Stromal Cells

**Figure 1:**
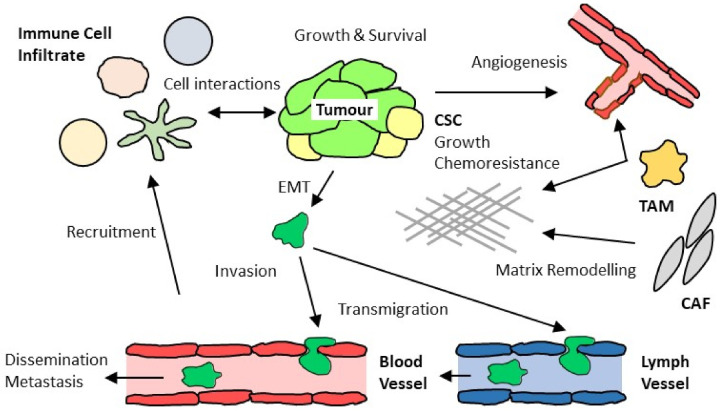
Complex roles of chemokines encompassing both tumor inhibiting and promoting functions. Chemokines are produced directly by tumor cells, tumor -associated vasculature, tissue macrophages and fibroblasts as well as recruited immune cells. Local chemokines shape tumor progression in many ways, often in synergy with other cytokines and metabolites. These include: recruitment of circulating immune cells and/or their retention in the tumor microenvironment; immune cells exerting pro- or anti-tumor immune responses; growth of tumors, including growth and chemoresistance of cancer stem cells; induction of epithelial–mesenchymal transition in tumor cells followed by their transmigration into lymphatic and/or blood vessels; dissemination of tumor cells to sites of secondary tumor growth; induction of angiogenesis; remodeling extracellular matrix by tumor-associated macrophages and cancer-associated fibroblasts. EMT, epithelial–mesenchymal transition; CSC, cancer stem cell; TAM, tumor-associated macrophage; CAF, cancer-associated fibroblast.

It is unclear if chemokines already play a role in the initiation phase of cancer, well before the development of macroscopic tumors. However, it is well documented that a plethora of chemokines affect cancer at later stages of diagnosis and treatment. I will briefly summarize recent progress in our understanding of how chemokines affect cancer development and refer the reader to a selection of excellent recent reviews that cover this topic [8,9,10,11,12,13,14,15]. I will then discuss in more detail the role of CCR8 in this process. Classical chemokine functions pertain to cell mobilization, and the mixture of chemokines reported to be present in tumors reflects both the dynamics and complexity of immune cells and stromal cells that define distinct stages of the disease (Figure 1). In fact, serum levels of certain chemokines may help define the cellular composition in solid tumors and may facilitate disease prognosis. The tumor microenvironment (TME) contains mobilized cells of both hematopoietic and tissue origin, including lymphocytes (αβ T cells, γδ T cells, NK T cells, B cells, NK cells) and myeloid cells (monocytes, macrophages, dendritic cells, neutrophils) as well as fibroblasts and endothelial cells recruited during tumor growth. Tumor tissue, like normal healthy organs, also contains macrophages of embryonic (non-hematopoietic) origin that respond to chemokines. Equally important, both stromal and immune cells are further subdivided into cells with distinct functions. Cancer-associated fibroblasts (CAFs) and tumor-associated macrophages (TAMs) are frequent residents of the tumor microenvironment and fulfil tumor supportive functions. αβ T cells consist of both immunosuppressive T cells (Treg cells) that promote tumor growth by inhibiting anti-tumor immune responses as well as anti-tumor T cells such as type-1 CD4^+^ Th cells and cytotoxic CD8^+^ T cells (CTLs) that are capable of recognizing and killing tumor cells. Of note, T cell subsets are distinguished by the combination of cell surface chemokine receptors, indicating that their mobilization is not governed solely by a single chemokine receptor axis [2,6,7]. This “redundancy” may account for lack of efficiency frequently observed when targeting single chemokine receptors in inflammatory diseases. Nevertheless, certain chemokine receptors stand out and merit special discussion.

The functional spectrum of chemokines present in tumors is in part context-dependent and combines pro- and anti-tumor features. It is difficult to single out distinct chemokine-chemokine receptor axes, as they frequently synergize with other chemokine systems as well as immunomodulatory cytokines, growth and differentiation factors. Consequently, chemokines are “moving” targets and need to be discussed together with the ever-changing tumor landscape. The following brief discussion exemplifies the most striking findings in cancer-related chemokine research; for in-depth information, the reader is referred to additional articles that accompany this Special Issue in Cancers entitled “Emerging Roles of Chemokines in Cancer Immunotherapy” as well as excellent recent reviews [8,9,10,11,12,13,14,15]. CXCR3 is frequently associated with type-1 immune responses, and its ligands CXCL9–11 are expressed in type-1 inflammatory conditions governed by IFN-γ. Since type-1 immune effector cells, including Th1 cells and cytotoxic CD8^+^ T cells, are the most effective anti-tumor immune cells, induction of the three CXCR3 ligands appears to be a valuable goal, possibly in combination with checkpoint blockage inhibitors (CBIs) that release the molecular brakes on tumor infiltrating lymphocytes (TILs). Of note, a “side-effect” of chronic IFN-γ exposure is the induction of PD-L1 in tumors, suggesting that IFN-γ may counteract anti-tumor T cell responses during long-term exposure to this cytokine [16,17]. Treg cells uniformly express CCR4, often in combination with receptors that specify the T helper cell counterpart they are selected to suppress, such as CXCR3 for Th1 cells, CCR6 for Th17 cells, or CCR9 for intestinal Th cells as well as corresponding cytotoxic CD8^+^ T cells. Additional immunosuppressive cells in the TME are tumor-associated macrophages (TAMs), myeloid-derived suppressor cells (MDSCs) and neutrophils. Notably, hematopoietic (monocyte-derived) macrophages, as opposed to embryonic (tissue-resident) macrophages, as well as tumor-induced MDSCs express CCR2 [8,15]. Inhibition of CCR2 will potentially neutralize a substantial immunosuppressive cell compartment in the TME, although beneficial (pro-inflammatory) DCs may also be negatively affected by this treatment. Neutrophils and neutrophilic MDSCs express CXCR2, and this pathway is considered an important target in ongoing clinical research. In addition to the inflammatory infiltrate, tumor cells themselves are targets for chemokines. Mobilized tumor cells, i.e., those that have undergone epithelial–mesenchymal transition (EMT), express chemokine receptors that promote their invasion, transendothelial migration and localization in secondary tissues, where they may differentiate into metastasis (Figure 1) [18,19]. Numerous studies highlight the importance of CXCR4 in tumor cell mobilization and metastasis to the bone marrow, where its single ligand CXCL12 is constitutively produced. Lymph node metastasis may involve CCR7, whose ligands CCL19 and CCL21 are expressed in lymph nodes and tumor-draining lymphatic vessels, respectively. Increasing evidence points to an important role for CCR9 in guiding mobilized, CCR9-expressing tumor cells to the gastrointestinal tract, notably the intestines, where its single ligand CCL25 is ubiquitously produced. Primary tumors themselves appear to “condition” distant sites through mechanisms that may involve the induction of chemokines for homing and survival of metastatic cells [19]. Additional chemokine-supported responses include tissue matrix remodeling via attraction of macrophages and fibroblasts as well as angiogenesis. Exciting new developments highlight the potential importance of atypical chemokine responses, i.e., those not directly related to cell recruitment and/or tissue retention [11,13,15,19]. These responses are achieved in synergy with chemokine-unrelated factors that affect distinct intracellular signaling pathways, including the WNT/b-catenin, the MEK/ERK or the PI3K/Act/mTOR pathways, leading to tumor cell growth, survival and EMT. It is worth noting that the TME is rich in chemokines, cytokines and growth factors whose function(s) when described in isolation (and often under in vitro conditions) often underestimates the quality and magnitude of pro- or anti-tumor responses induced in combination with other stimuli. In summary, it is safe to say that tumor immune surveillance and disease progression greatly depend on chemokines whose functional repertoire spans both chemokine prototypic (migration) and atypic (function modulation) cell responses. The following sections focus on the inhibitory role(s) of Treg cells in anti-tumor immunity and conclude with a discussion about chemokine-based strategies that may target their presence in tumors.

## 3. Treg Cell-Targeted Therapies

Treg cells are the major cellular compartment tasked with the suppression of excessive (or inappropriate) T and B cell responses that may cause tissue damage if left unchecked [20,21,22,23,24]. Treg cells are highly diverse in terms of differentiation and activation stages and arise either from T cell precursors in the thymus (tTregs) or naïve CD4^+^ T cells initiated during antigen-priming in secondary lymphoid tissues (iTregs). tTreg cells recognize self-antigens and primarily reside in peripheral tissues, where they inhibit Tconv cells recognizing local self-antigens, whereas iTreg cells probably provide a negative feedback loop for effector Tconv cells to prevent excessive inflammatory responses in target tissues. In healthy individuals, Treg cells constitute a minor subset of 1–4% among CD4^+^ T cells in blood and peripheral tissues, but their numbers are known to increase in chronic diseases. In cancer, they vary in numbers but generally represent the major immunosuppressive force. A recent CyTOF protein expression study, involving 32 patients with pancreatic cancer, revealed a frequency of 8 to >30% Treg cells present among CD4^+^ T cells extracted from tumor tissue, of which the majority were activated and exhibited strong suppressive activity [25].

The identification of Treg cells in the context of cancer dates back many years. In fact, the concept of T cell-mediated suppression of anti-tumor immunity was first described 40 years ago by Berendt and colleagues [26]. The field quickly moved forward thanks to two seminal findings: the discovery of CD25 as a cell surface marker distinguishing Treg cells from resting CD4^+^ T conv cells [27], and Forkhead box P3 (FoxP3), the master transcription factor essential for Treg cell function [28,29,30]. Lack of FoxP3 in mice prevented the development of Treg cells and caused severe autoimmune disease, and forced FoxP3 expression turned Tconv cells into suppressive T cells [20]. FoxP3 is also transiently expressed in activated Tconv cells, which may cause problems in identifying bona fide Treg cells in human diseases, including cancer [21,22].

Thanks to new technologies, including single cell RNA sequencing (scRNAseq) and mass cytometry (CyTOF), we now have a better understanding of the phenotype and tissue distribution of Treg cells in both mice and humans. In human tumors, canonical cell surface markers of Treg cells include immune checkpoints CTLA-4, PD-1, TIM3 and LAG3, and the co-stimulatory receptors GITR, ICOS and OX40 [21,31,32], and their level of expression correlates with the state of Treg cell activation and suppressive function. Single cell analyses further revealed additional characteristics linking tumor Treg cells with their counterparts in healthy tissues, including the transcription factors BATF, Relb and Ikzf2 that contribute to Treg cell specialization during their migration from secondary lymphoid tissues into their target tissue [33,34]. A fraction of tumor Tconv cells share makers, including CD103 and CD69, with tissue-resident memory T (Trm) cells, a subset of non-circulatory memory T cells comprising the first line of adaptive defense in healthy tissues [35,36]. However, TCR repertoire analyses of Treg cells in patients with metastatic melanoma showed a significant overlap between tumor tissue and blood compartments, indicating that their tissue-residency is not stringent [37]. In fact, the whole concept of tissue-residency has recently been revised since human and mouse Trm cells themselves displayed some degree of circulatory behavior that was linked to their level of activation [38,39]. These exciting findings are relevant to the present discussion for two reasons: first, it appears possible to improve anti-tumor immunity by targeting circulating T cells, i.e., tumor-specific T cells found in blood and, second, changes in the composition of circulating T cells during cancer immunotherapy may be used as a correlate for the response to treatment.

**Figure 2:**
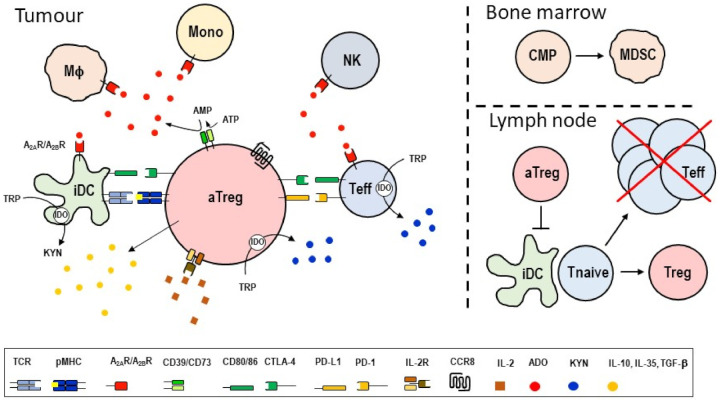
Tumor Treg cells elaborate diverse immunosuppressive functions affecting local (tumor-resident) and distal (lymphoid tissue-resident) immune cells. Tumor Treg cells also activate local tissue cells (not shown here). At the tumor site, activated Treg cells directly engage with conventional effector T cells and DCs. Enzymatic conversion of extracellular metabolites (ATP, tryptophan) leads to the production of inhibitors that suppress the function of effector T cells, NK cells, macrophages and monocytes. Changes to the cytokine milieu (depletion of IL-2 and production of TGF-β, IL-13 and IL-35) further augments the immunosuppressive function of Treg cells. In addition to local effects, both tumor Treg cells and their soluble products reach lymphoid tissues, including tumor draining LNs and bone marrow, where these factors amplify the immunosuppressive conditions by inhibiting anti-tumor effector T cells while generating additional Treg cells and myeloid-derived suppressor cells. aTreg, activated Treg cell; Teff, effector T cell; Tnaive, naïve T cells; iDC, inhibitory DC; NK, NK cell; MΦ, macrophage; CMP, common myeloid progenitor cell; Mono, monocyte; ADO, adenosine; IDO, indoleamine 2,3-dioxygenase, TRP, tryptophane; KYN, N-formyl-kynurenine.

Tumor Treg cells suppress anti-tumor immune responses by a myriad of mechanisms (Figure 2). CTLA-4 binds with high affinity to the co-stimulatory ligands CD80 and CD86 on dendritic cells (DCs), thereby blocking the stimulatory signals mediated by CD28 that are essential for T cell differentiation during primary immune responses [40]. CD25 is the α-chain of the trimeric, high-affinity IL-2 receptor, and its overexpression on Treg cells causes the depletion of extracellular IL-2 necessary for growth and expansion of Tconv cells [41]. Treg cells also express the ectonucleotidases CD39 and CD73 that convert ATP into adenosine. Triggering of the A2aR/A2bR adenosine receptors results in the elevation of intracellular cAMP and subsequent immune suppression in Tconv cells, including inhibition of TCR signaling and cell migration [42,43]. Cell surface indoleamine 2,3-dioxygenase (IDO) contributes to the depletion of extracellular tryptophane and leads to the production of inhibitory metabolites, including N-formyl-kynurenine, that also interfere with T cell activation [44]. Furthermore, activated Treg cells secrete IL-10, IL-35 and TGF-β and other immunosuppressive cytokines that act locally on immune and stromal cells or suppress Tconv cell differentiation in tumor-draining lymph nodes. Of interest, Treg cells themselves express PD-L1 and, thus, may directly inhibit tumor Tconv cells via binding to PD-1, causing tyrosine phosphatase SHP-2-mediated inhibition of TCR signaling [45]. The impressive inventory of immune suppressive mechanisms displayed by tumor Treg cells may act locally in the TME as well as distally in the tumor-draining lymph nodes (Figure 2).

For the reasons discussed above, tumor Treg cells have become a prime target in translational research, culminating in several CTLA-4-inhibitory antibodies (ipilimumab, tremelimumab) currently approved for use in immunotherapy of patients with solid cancers [16,17,46,47]. Similarly, a second class of approved antibodies target PD-1 (pembrolizumab, nivolumab, cemiplimab), a second checkpoint receptor or its ligand PD-L1 (atezolizumab, avelumab, durvalumab), which are thought to augment anti-tumor immunity by reinvigorating PD-1-expressing, exhausted tumor-specific CD8^+^ T cells [17,46,47]. However, the mechanism of action of PD-1/PD-L1-specific antibodies may go well beyond CD8^+^ T cells and may include PD-1-expressing myeloid cells, as recently demonstrated in mouse tumor models [48]. A firm understanding of the type and tissue distribution of immune cells expressing one or both of these checkpoint inhibitors is of paramount importance for the management of immune-related adverse events (irAEs) frequently observed in patients undergoing CBI immunotherapy (see below).

Recently, two reports described the selective expression of the chemokine receptor CCR8 on tumor-associated Treg cells recovered from patients with breast cancer (BC), non-small cell lung cancer (NSCLC), colorectal cancer (CRC) and melanoma (ME) [49,50]. Subsequent global gene and protein expression studies confirmed and extended these findings to additional solid tumors, both in humans [51,52,53,54] and in mice [55,56,57,58,59]. The discussions in the following chapters are focused on the chemokine receptor CCR8 and the prospect for success of CCR8-targeted therapies in patients with solid cancer.

## 4. Brief History of CC Chemokine Receptor 8 (CCR8)

Future cancer-targeted therapies may benefit from a more detailed understanding of the involvement of CCR8 and its ligands in immunological processes. Human CCR8 was identified in 1997, either by means of low-stringency PCR screening [60] or by means of functional screening of orphan GPCRs [61]. Human CCR8 has two selective ligands, CCL1, reported in 1989 as a secreted, T cell-derived protein [62] and CCL18, described 25 years later [63]. Human and mouse CCR8 are structurally related [64] and so is their primary ligand CCL1 [65]. The second ligand for mouse CCR8 is CCL8, a high affinity chemokine that differs from human CCL18 both in terms of structural similarity and superior functional potency [63,66]. Considering the rapid pace of chemokine research, studies of CCR8 were hindered by two early observations, (1) the scarcity of CCR8 on immune cells in human peripheral blood, and (2) the lack of striking phenotypic abnormalities in *ccr8*-targeted mouse models. Despite early conflicting reports [67,68,69], recent in vivo studies revealed a clear role for CCR8 in type-2 inflammatory diseases, including atopic dermatitis [66,70] and allergic enteritis [71]. It is not clear at present, however, whether the findings in mice also translate to human inflammatory diseases. Furthermore, the CCR8/CCL1 axis plays a non-redundant role in cutaneous DC traffic in mice [72,73,74,75], yet CCR8 was not detected on human skin DCs. In addition, much more is known about the cellular sources of CCL1 than the two more recent ligands, mouse CCL8 and human CCL18. In agreement with earlier reports, CCL1 is primarily a T cell product [65,76,77,78,79] and, in addition, is also secreted by tissue phagocytes (macrophages, DCs), mast cells and tumor cells.

**Figure 3:**
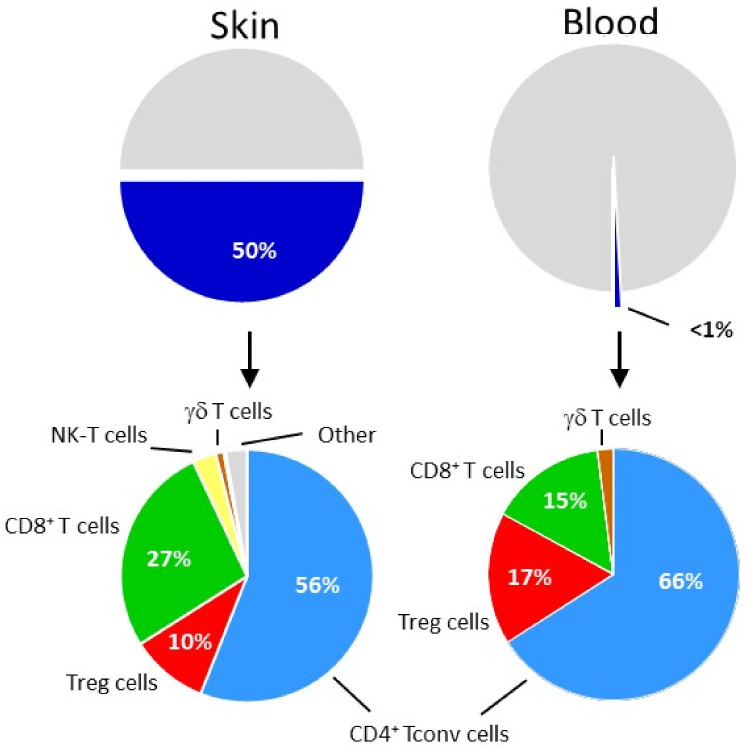
Human skin is the principal site of CCR8-expressing lymphocytes and NK cells. Protein expression analyses demonstrate a predominance of CCR8^+^ cells among lymphocytes, amounting to 50% of all immune cells present in human skin. Of these, CD4^+^ and CD8^+^ conventional αβ T cells and Treg cells make up >90%. CCR8^+^ immune cells are rare in peripheral blood and share the subset diversity with human skin CCR8^+^ immune cells (except for NK cells). Numbers represent fractions of CCR8^+^ cells expressed as the mean percentage of total lymphoid cells (T and B cells, ILCs) out of *n* = 3–12 independent experiments with skin tissue or blood samples from >20 individual donors ([77,78,79] and unpublished studies).

Advanced staining reagents greatly accelerated human CCR8 research and refined early reports describing CCR8 expression in human blood Th2 and Treg cells (Figure 3; Table 1) [80,81,82,83]. Indeed, fluorescently labelled CCL1 [84,85] and CCR8-specific antibodies [77,78] provided the means to definitely define the identity of CCR8^+^ cells among PBMC, confirming their relative scarcity in blood. The vast majority of CCR8^+^ cells are found among lymphocytes, especially among CD4^+^ αβ T cells, including FoxP3^+^ Treg cells and memory Tconv cells. CCR8 was absent on any other type of immune cell, except for a few γδ T cells and NK cells (see below). Interestingly, human blood monocytes were originally reported to respond to CCL1/I-309 [86] and to express CCR8 mRNA [60]; however, these early findings were not corroborated by subsequent CCR8 protein expression studies [77,84]. In fact, healthy human skin turned out to be the primary residence of CCR8^+^ cells [76,77,78]. The diversity of CCR8^+^ cells among skin immune cells mirrored the situation in peripheral blood (Figure 3). However, and in clear contrast to blood, the total number of CCR8^+^ cells within healthy skin tissue far exceeded the number of CCR8^+^ cells present in peripheral blood, with 50% of all skin αβ T cells (equivalent to approx. 10^10^ cells) expressing this chemokine receptor. CCR8 designates “skin-homing” T cells since circulating and skin-resident CCR8^+^ T cells co-express the cutaneous T cell antigen (CLA), an adhesion receptor known to guide immune cells into skin tissue. The small fraction of CCR8^+^ γδ T cells are Vδ1^+^ γδ T cells. The γδ T cell subset featuring the Vδ1-chain in their TCR is rare in blood but predominates in human skin. Similarly, CCR8 is also found on CD16-expressing NK cells that reside primarily in human skin but not in blood [76,77]. However, αβ T cells within healthy skin are by far the largest fraction of CCR8-expressing immune cells, suggesting that therapeutic considerations should be focused primarily on these cells. A “skinness” of the CCR8-axis is also inferred from the skin-tropic poxvirus molluscum contagiosum whose CCR8-specific antagonist, MC148, is expressed early during infection and may help to evade anti-viral immunity by inhibiting cutaneous CCR8^+^ Tconv cells [87]. In support of their skin-selective residency, CCR8^+^ T cells are largely absent in other human tissues, as evidenced by recent RNA expression studies [49,50].

Human skin CCR8^+^ T cells bear many hallmarks of resident memory T (Trm) cells [88,89,90], including CD69/CD103 expression, steady-state proliferation in response to local growth factors IL-7 and IL-15, and lower expression of transcription factors Eomes and T-bet [79]. In contrast, skin T cells lacking CCR8 were more variable in phenotypic and functional markers, expressed higher levels of inhibitory receptors, including PD-1, as well as senescence markers CD57 and KLRG1 and showed poor proliferative responses ex vivo. The two skin T cell compartments distinguished by CCR8 expression may have arisen from separate (unrelated) antigenic challenges, as evidenced by TCR Vβ clonotype analyses [79]. Based on mouse in vivo studies, CCR8 is dispensable for the generation of cutaneous Trm cells [91], suggesting a more local role for CCR8 in skin immune surveillance.

## 5. Relationship between Humans Skin and Tumor Treg Cells

Skin Treg cells uniformly (>85%) express CCR8 as opposed to conventional skin CD4^+^ and CD8^+^ αβ T cells with 56 ± 18% and 27 ± 18% being positive for CCR8, respectively (Figure 3) [77,78,79]. The fact that human skin and tumor Treg cells share the unique feature of expressing CCR8 suggests similar mechanisms underlying the control of CCR8 gene expression. In vitro studies with naïve human CD4^+^ T cells have revealed the importance of the tissue environment in this process (reviewed in [3]). Skin tissue-derived soluble factors, notably 1,25-dihydroxyvitamin D3 (the active metabolite of vitamin D3) and prostaglandin E2, were highly efficient inducers of CCR8 (as well as CLA) in TCR-stimulated CD4^+^ T cells [78,100]. The two components, skin factors and TCR triggering, were acting in concert, since they did not induce CCR8 expression on their own. The situation resembles the induction of a gut-homing phenotype, defined by co-expression of CCR9 and a4b7 in T cells, which requires the involvement of the vitamin A metabolite, all-trans retinoic acid (reviewed in [101]). Both types of vitamins act via heterodimeric nuclear receptors composed of retinoic-X receptor (RXR) in combination with either vitamin D or retinoic acid receptor, respectively. It is possible that the tissue microenvironment plays a similar role in the localization of Treg cells elsewhere in the body [102,103,104] (reviewed in [105]). CCR8 signaling by itself was shown to enhance the level of cell surface CCR8 in Treg cells [106]. In analogy to human skin T cells [3], I propose that solid tumors mimic skin by providing CCR8-inducing factors necessary for induction of CCR8 in tumor Treg cells [107,108,109,110,111].

Co-expression of CCR8 may also suggest overlapping functionalities in skin and tumor Treg cells that may go well beyond local immunosuppression. Obvious similarities between healthy skin and tumors are their regenerative or wound-healing capabilities. In fact, the concept that tumors are “wounds that do not heal” was proposed many decades ago (reviewed in [112]), and recent global gene expression analyses support this view by assigning wound-healing gene expression signatures to tumor-associated Treg cells in multiple solid human cancers [113]. Furthermore, epigenetic and gene expression analyses clearly link human skin Treg cells with CCR8 and the transcription factor BATF, presumed to be a critical factor for the induction of a tissue repair program [33,114]. Of interest, visceral adipose tissue (VAT) Treg cells share these features with skin Treg cells; by contrast, intestinal Treg cells are not hardwired to express CCR8 and BATF, suggesting that the functional specialization of Treg cells is aligned with discrete tissue-homing properties [113]. In mice, VAT Treg cells regulate metabolic pathways [115,116,117], whereas skin Treg cells contribute to tissue homeostasis and repair [118,119]. Similarly, the presence of “tissue repair” Treg cells, distinguished by co-expression of KLRG1, amphiregulin and CCR8 in a mouse lung adenocarcinoma model, suggests conserved Treg cell differentiation pathways in advanced solid tumors [120]. Collectively, the continuous need for tissue repair in healthy skin and solid tumors may have led to the establishment of specialized Treg cells featuring CCR8 expression in combination with powerful wound healing and immune suppressive functions. One may speculate that interference with CCR8^+^ tumor Treg cells could not only reduce suppression of anti-tumor immunity but also impair tumor repair mechanisms, which is another potentially beneficial effect of CCR8-targeted immunotherapy.

## 6. CCR8 Marks Activated Intratumoral Treg Cells

Tumor-infiltrating FoxP3^+^ Treg cells are heterogenous in terms of phenotype and function [25]. Of note, CCR8 expression marks highly active Treg cells featuring the highest levels of inhibitory receptors, including CTLA-4, TGIT, and co-stimulatory receptors ICOS, CD30 and 4-1BB, whose superior immunosuppressive function is largely governed by TCR signaling in combination with the transcription factors BATF and IRF4 [34,49,50,53,121,122]. Equally important, the fraction of peripheral blood CCR8^+^ Treg cells (approx. 40% of all FoxP3^+^ Treg cells) are phenotypically similar to tumor CCR8^+^ Treg cells and share many TCR clonotypes with tumor CCR8^+^ Treg cells [37,51,52]. Intratumoral Treg cells recognize tumor antigens, including neoantigens [37]. These findings suggest that tumor-specific Treg cells recirculate between the tumor tissue and blood compartments, providing a rationale for targeting CCR8 on circulating Treg cells. In vivo evidence in favor of studying CCR8 has been reported very recently by several groups. Preclinical studies in mice modelling colon (BC38, CT26), melanoma (B16F10), breast (EMT6) and urothelial (MB49) cancer revealed that tumor Treg cells expressed high levels of CCR8 whose targeting in monotherapies with mouse CCR8-specific antibodies resulted in significant inhibition of tumor growth, equivalent to what has been seen with anti-PD-1 blocking antibodies [55,56,57,58,59]. The primary mode of action was reported to involve antibody-dependent cellular cytotoxicity (ADCC) leading to the elimination of CCR8-expressing tumor Treg cells [56,58]. Combination therapies using antibodies specific for both CCR8 and PD-1 led to near complete arrest in tumor progression, indicating that these reagents work in synergy. It will be interesting to examine alternative combination therapies, such as those combining anti-CCR8 antibodies with tumor vaccines or MDSC-targeted therapies. 

The physiological significance of CCR8 on tumor Treg cells is not clear at present but, based on tumor models in mice, appears unlikely to be associated with the recruitment of tumor Tregs cells. Still, it is worth mentioning that CCL18, the second ligand for human CCR8, is strongly expressed in patients with solid tumors and reported to promote tumor progression by different means, including Treg cell and type-2 macrophage differentiation as well as mobilization of tumor cells (reviewed in [123]. It is, therefore, possible that CCL18 contributes to the presence of CCR8^+^ Treg cells within tumors. Unfortunately, mechanistic studies are hampered by the fact that CCL18 is known to interact with 3 different cell surface receptors, PITPNM3, GPR30 and CCR8. Similarly, absence of a mouse orthologue makes in vivo studies of CCL18 more challenging.

## 7. Safety Concerns Related to CCR8-Targeted Therapies

Collectively, CCR8 identifies tumor Treg cells actively engaged in the suppression of anti-tumor immunity by a combination of mechanisms as summarized in Figure 2. Tconv cells within tumors as well as adjacent (healthy) tissue do not express CCR8. In fact, the selectivity of CCR8 for intratumoral Treg cells is remarkable and warrants a more detailed discussion about potential safety concerns vis-à-vis approved antibody-based drugs that are widely used in current cancer therapies. CBI antibodies targeting CTLA-4 and the PD-1/PD-L1 pathways have dramatically advanced the treatment success in many patients with solid cancer [17,46]. Inflammatory toxicities, including barrier tissue injuries and organ dysfunctions, frequently necessitate interruption or even termination of ongoing therapy in affected patients with cancer [124,125]. Fatalities due to myocarditis, endocrine dysfunction or colitis were also reported in a low number of patients receiving combination therapies [126]. Neither CTLA-4 nor PD-1 are selectively expressed in tumors; in fact, these receptors are indispensable for controlling adaptive immune responses, either in a negative feedback loop during ongoing T cell responses or during tissue homeostasis by inhibiting auto-reactive effector T cells [127]. Therefore, it is feasible that aberrant autoimmune manifestations and subsequent tissue damage observed during prolonged anti-CTLA-4/PD-1 treatment may have resulted from releasing the “breaks” on autoimmune T cells present within healthy peripheral tissues and organs. 

Mogamulizumab, a non-fucosylated, humanized antibody specific for human CCR4, is another recent example of an approved antibody reagent causing irAEs in patients with cancer [128,129]. The ADCC-active antibody reagent is approved in the USA and Europe for the treatment of cutaneous T cell lymphomas, including patients suffering from advanced forms of mycosis fungoides or Sezary syndrome, as well as in Japan for advanced adult and peripheral T cell lymphoma [130,131,132]. The distribution of CCR4^+^ cells is relatively widespread and includes platelets, NK cells, mast cells and T cells [4]. Relevant to the current discussion is the global expression of CCR4 on Th2 cells typically found in type-2 inflammatory diseases, e.g., allergic asthma and atopic dermatitis, and Treg cells with essential immune surveillance functions in health tissues/organs. Considering the relative broad tissue distribution of CCR4^+^ T cells (including Treg cells in healthy tissues), it is interesting to note that severe irAEs in T cell lymphoma patients undergoing mogamulizumab treatment were rarely observed (20% of all patients showed adverse reactions, of which 36% were severe) [133]. 

In comparison to CCR4, T cells expressing CCR8 are much less common, being primarily restricted to skin T cells (Figure 3; Table 1) and tumor Treg cells (as discussed above). Additional CCR8^+^ cells (other than lymphocytes), as summarized in Table 1, are often not detected by flow cytometry or represent minor immune cell subsets that may not affect the outcome of future CCR8-directed therapies. Based on these considerations, it is unlikely that CCR8-specific antibodies will cause irAEs exceeding those observed in patients treated with mogamulizumab (see review article by O. Yoshie in this Special Issue in Cancers). 

## 8. Conclusions

As suggested by the recent data from several mouse cancer models [55,56,57,58,59], CCR8 represents a promising target for cancer immunotherapy. Following the lead of mogamulizumab, ADCC-active antibodies to CCR8 could bind to and eliminate tumor-associated Treg cells, notably the most activated and suppressive fraction of Treg cells that selectively express this chemokine receptor. Resting (CCR8-negative) tumor-associated Treg cells are probably not actively engaged in suppressing anti-tumor immunity and would not be affected by this treatment. The holy grail in cancer research is the identification of tumor-specific targets whose interference induces minimal irAEs. In this respect, CCR8 represents an exciting option, since its expression is minimal in beneficial (helper/cytotoxic) T cells in the TME as well as any type of T cell in adjacent non-affected tissues. In this respect, CCR8 represents a more tumor Treg cell-specific target than CCR4, which is known to be broadly expressed among blood and tissue Treg cells. The obvious caveat is human skin, the primary reservoir of CCR8^+^ cells, which would require special management during CCR8-targeted therapies. Ultimately, this hypothesis will need to be tested in clinical trials involving patients with solid tumors.

## Figures and Tables

**Table 1:** Cellular distribution of human CCR8.

Tissue/Cells	Comments	Refs
Blood Tregs cells	Subset of CD4^+^CD25^+^ T cells (IHC ^1^) that co-express CCR4 and CLA FC ^2^ data with CCL1-AF or antibody reagents confirm that 40% of blood FoxP3^+^ Treg cells express CCR8 CCR8^+^ Treg cells co-express CCR4 and skin-homing receptor CLA In vitro chemotaxis to CCL1	[77,80,82,84]
Skin Treg cells	Majority (>90%) of cutaneous FoxP3^+^ Treg cells express CCR8 and co-express CCR4 and CLA	[79]
Blood Tconv cells	Original data with activated Th2 cells (NB ^3^) FC data with CCL1-AF or antibody reagents demonstrate minor (<20%) fraction of Tconv cells that express CCR8 (FC) CCR8^+^CD4^+^ Tconv cells outnumber CCR8^+^CD8^+^ T cells by 4:1 (FC) CCR8 expressed on minor fraction of Vδ1^+^ γδ T cells, whereas dominant fraction of Vδ2^+^ γδ T cells lack CCR8 (FC)	[76,77,78,81,84]
Skin Tconv cells	Half of all cutaneous Tconv cells express CCR8; of these, CD4^+^ T cells outnumber CD8^+^ T cells by 2:1 (FC) Co-expression of CCR4, CXCR3 and skin homing receptor CLA (FC) Diverse cytokine expression profile by CCR8^+^ Tconv cells (FC) Vδ1^+^ γδ T cells express CCR8 (Vδ2^+^ γδ T cells are not present in skin) (FC)	[76,77,78,79,84]
Blood NK cells	Activated NK lines express CCR8 (NB, FC) and respond to CCL1 (chemotaxis) CCR8 expression rare on blood NK cells (FC)	[76,84,92,93]
Skin NK cells	CCR8 expression on cutaneous CD56^+^CD16^−^ NK cells (FC); CD56^+^CD16^+^ fraction of NK cells, which predominate in blood, lack CCR8 (FC)	[76,79]
Thymocytes	CD4^+^CD25^+^ thymocytes express CCR8 (NB, FC) and migrate in response to CCL1	[94,95,96]
Endothelial cells	Aortic endothelial cells express CCR8 (IHC) Human vascular endothelial cell line migrates in response to CCL1	[97,98]
Cancer	Human adult T cell leukemia express CCR8 (NB) and migrate in response to CCL1 Activated Treg cells in solid tumors express CCR8 (RNASeq ^4^, FC), and migrate in response to CCL1	[49,50,51,52,53,54,99]

Methods for detection of human CCR8: ^1^ IHC, immunohistochemistry; ^2^ FC, flow cytometry; ^3^ NB, Northern blot; ^4^ RNASeq, global single-cell RNA expression analyses.

## Data Availability

Not applicable.

## Abbreviations

CCR8	CC chemokine receptor 8
CTLA-4	cytotoxic T lymphocyte associated protein-4
PD-1/PD-L1	program death receptor-1/ program death receptor-1 ligand-1
irAEs	immune-related adverse events
CBIs	checkpoint blockade inhibitors
Treg/aTreg	regulatory T cells/activated regulatory T cells
Tconv	conventional T cell
Teff	effector T cell
NK	natural killer cell
DC/iDC	dendritic cell/inhibitory dendritic cell
MDSC	myeloid derived suppressor cell
Mf	macrophage
CAF	cancer associated fibroblast
TAM	tumor associated macrophage
IFN-g	interferon gamma
TNF-a	tumor necrosis factor alpha
TGF-b	transforming growth factor beta
TRP	tryptophane
KYN	N-formyl-kynurenine
IDO	indoleamine 2,3-dioxygenase
EMT	epithelial mesenchymal transition
CSC	cancer stem cell
